# Whole-genome sequencing of *Listeria monocytogenes* isolated from the first listeriosis foodborne outbreak in South Korea

**DOI:** 10.3389/fmicb.2023.1182090

**Published:** 2023-06-02

**Authors:** Seung Hun Lee, Sangmi Lee, Sang Hun Park, Ok Kyung Koo

**Affiliations:** ^1^Department of Food and Nutrition, Gyeongsang National University, Jinju, Republic of Korea; ^2^Department of Food and Nutrition, Chungbuk National University, Cheongju, Republic of Korea; ^3^Seoul Metropolitan Government Research Institute of Public Health and Environment, Seoul, Republic of Korea; ^4^Department of Food Science and Technology, Chungnam National University, Daejeon, Republic of Korea

**Keywords:** *Listeria monocytogenes*, foodborne outbreak, South Korea, WGS, MLST, *llsX*

## Abstract

*Listeria monocytogenes* is a foodborne pathogen that causes listeriosis in humans with severe symptoms. In South Korea, listeriosis had only been reported sporadically among hospitalized patients until the first foodborne outbreak occurred in 2018. In this study, a *L. monocytogenes* strain responsible for this outbreak (FSCNU0110) was characterized via whole genome sequencing and compared with publicly available *L. monocytogenes* genomes of the same clonal complex (CC). Strain FSCNU0110 belonged to multilocus sequence typing (MLST)-based sequence type 224 and CC224, and core genome MLST-based sublineage 6,178. The strain harbored tetracycline resistance gene *tetM*, four other antibiotic resistance genes, and 64 virulence genes, including *Listeria* pathogenicity island 1 (LIPI-1) and LIPI-3. Interestingly, *llsX* in LIPI-3 exhibited a characteristic SNP (deletion of A in position 4, resulting in a premature stop codon) that was missing among all CC224 strains isolated overseas but was conserved among those from South Korea. In addition, the *tetM* gene was also detected only in a subset of CC224 strains from South Korea. These findings will provide an essential basis for assessing the characteristics of CC224 strains in South Korea that have shown a potential to cause listeriosis outbreaks.

## Introduction

Although globalization has provided opportunities for consumers to enjoy a wide range of products and expanded global food trade, the complexity of the international food supply has contributed to an increase in foodborne outbreaks (Quested et al., 2010; Hussain and Dawson, 2013). Worldwide efforts have ensured food safety by employing advanced hygiene management technology such as the hazard analysis critical control point (HACCP) system (Weinroth et al., 2018). However, despite such efforts, foodborne pathogens are still causing a large number of diseases and deaths every year (WHO, 2022).

*Listeria monocytogenes* is a gram-positive facultative foodborne pathogen that causes listeriosis in humans (Swaminathan and Gerner-Smidt, 2007; Varma et al., 2007). Listeriosis has a significant mortality rate (20 ~ 30%) and mainly affects people with weakened immune systems, such as pregnant women, newborns, the elderly, and immune-compromised patients (CDC, 2016). Approximately 2,000 cases of listeriosis and 200 deaths are reported every year in 27 countries of the European Union (EFSA and ECDC, 2021). In the United States, *L. monocytogenes* is estimated to be responsible for approximately 1,600 cases and 260 deaths annually (CDC, 2016). *L. monocytogenes* is widely distributed in natural environments, processing facilities, and food products (Farber and Peterkin, 1991). These characteristics, i.e., the ubiquitous presence and high mortality rate, have made *L. monocytogenes* as one of the major threats to the food industry and public health (Beresford et al., 2001).

*Listeria monocytogenes* has been classified into four lineages and 13 serotypes that are grouped into four PCR serogroups (Doumith et al., 2004). Notably, serotypes 1/2a, 1/2b, and 4b have caused most human listeriosis cases (Kramarenko et al., 2013; Maury et al., 2016). In the meantime, multilocus sequence typing (MLST) has been utilized as a standard genotyping method to compare distinct clonal groups of *L. monocytogenes* with regard to the differences in the nucleotide sequences of the housekeeping genes (Moura et al., 2016). Whole genome sequencing (WGS) has recently been used for subtyping *L. monocytogenes* strains as exemplified by the core genome MLST (cgMLST) that compares the sequence differences of 1,748 core genes and detect virulence and antibiotic resistance genes to assist in the epidemiological investigation into the source of the disease (Moura et al., 2016; Gautam et al., 2019).

Over 100 foodborne outbreaks in South Korea are caused every year by pathogenic bacteria such as *Salmonella*, pathogenic *Escherichia coli*, and *Campylobacter jejuni* (MFDS, 2022). However, the first foodborne outbreak caused by *L. monocytogenes* in South Korea occurred in 2018 (Han et al., 2019). In this outbreak, 294 people experienced listeriosis symptoms after lunch served at a school cafeteria, and 64 were confirmed to be infected with *L. monocytogenes* (Han et al., 2019). *L. monocytogenes* was isolated from seasoned crab meat with bean sprouts (served at lunch), spicy cold noodles and green pudding salad (both served at dinner). A retrospective cohort study and PFGE analysis showed that seasoned crab meat with bean sprouts was significantly associated with the disease (Han et al., 2019). Although listeriosis has been uncommon in Korea, the incidence of listeriosis among the elderly and immunocompromised patients in three Korean hospitals increased from 1.8 per 100,000 inpatients in 2006–2012 to 5.5 in 2013–2016 (Choi et al., 2018). There has also been a recent case of pregnancy-related listeriosis, resulting in premature infant death (Yun et al., 2020). To deepen our understanding of the genetic features of the outbreak strain and other *L. monocytogenes* strains isolated from South Korea, this study aimed to obtain the whole genome sequence of a strain isolated from a patient afflicted by this outbreak and to compare it with food isolates collected in South Korea. In addition, this study compared those obtained overseas with regard to MLST and cgMLST profiles and genes involved in virulence and antibiotic resistance.

## Materials and methods

### Bacterial strains and DNA isolation

*Listeria monocytogenes* strains isolated from the aforementioned listeriosis outbreak (FSCNU0110) and foods sold in South Korea between 2005 and 2021 were used for serotyping, MLST, and antibiotic resistance assays (Table 1). Strain FSCNU0110 was isolated from feces of a patient who experienced two or more episodes of fever, abdominal pain, diarrhea, and vomiting after consuming food in the school cafeteria (Han et al., 2019). Bacterial cells were plated on brain heart infusion (BHI) agar (MB cell, Seoul, South Korea) and incubated at 35 ± 1°C for 18–24 h. Colonies on the BHI plate were confirmed as *L. monocytogenes* using the VITEK-2 automated microbial identification system (BioMérieux, Marcy-l’Etoile, France). Isolates were stored as 15% glycerol stock at −80°C until use. Genomic DNA was extracted using the AccuPrep^®^ Genomic DNA Extraction kit (Bioneer, Daejeon, South Korea) to designate PCR-serogroup, and MLST-based sequence type (ST) and clonal complex (CC). The quantity and quality of genomic DNA were measured using Nanodrop one (Thermo Fisher Scientific, Waltham, MA, United States), and the genomic DNA was stored at −20°C until further use.

**Table 1:** Characteristics of *Listeria monocytogenes* isolates from South Korea analyzed in this study.

Strain	Source	Year	Serotype	PCR-serogroups	Lineage	ST	References
FSCNU0002	Seasoned pork	2005	1/2c	llc	ll	9	This study
FSCNU0003	Seasoned pork	2005	1/2b	llb	l	224	This study
FSCNU0007	Seasoned pork	2005	1/2c	llc	ll	9	This study
FSCNU0045	Seasoned pork	2005	1/2b	llb	l	224	This study
FSCNU0052	Seasoned pork	2006	1/2c	llc	ll	9	This study
FSCNU0057	Seasoned pork	2006	1/2a	lla	ll	37	This study
FSCNU0060	Seasoned raw crab	2006	1/2b	llb	l	87	This study
FSCNU0062	Seasoned raw crab	2006	1/2b	llb	l	3	This study
FSCNU0066	Seasoned pork	2007	1/2a	lla	ll	101	This study
FSCNU0089	Seasoned pork	2005	1/2c	llc	ll	9	This study
FSCNU0093	Seasoned pork	2005	1/2b	llb	l	87	This study
FSCNU0095	Korean style raw beef	2010	4b	IVb	l	2	Park et al., 2016
FSCNU0098	Slices of boiled meat	2010	1/2b	llb	l	88	Park et al., 2016
FSCNU0099	Smoked salmon	2010	1/2a	lla	ll	121	Park et al., 2016
FSCNU0100	Seasoned dried squid	2010	1/2a	lla	ll	7	Park et al., 2016
FSCNU0110^a^	Human	2018	1/2b	llb	l	224	Han et al., 2019
FSCNU0111^a^	Human	2018	1/2b	llb	l	224	Han et al., 2019
FSCNU0113^a^	Human	2018	1/2b	llb	l	224	Han et al., 2019
FSCNU0114^a^	Human	2018	1/2b	llb	l	224	Han et al., 2019
FSCNU0115^a^	Seasoned crab meat with bean sprouts	2018	1/2b	llb	l	224	Han et al., 2019
FSCNU0120	Beef	2021	1/2b	llb	l	3	Lee et al., 2022
FSCNU0121	Beef	2021	1/2a	lla	ll	8	Lee et al., 2022
FSCNU0122	Beef	2021	1/2b	llb	l	5	Lee et al., 2022
NCCP14714	Human	2009	1/2b	llb	l	2,929	Type strain
NCCP15743	Human	2012	1/2a	lla	ll	101	Type strain

^a^Outbreak strain.

### Antisera serotype determination

The serotypes of *L. monocytogenes* isolates were obtained using *Listeria* antisera (Denka Seiken Co., Tokyo, Japan) according to the manufacturer’s instructions. To illustrate, *L*. *monocytogenes* was incubated on BHI agar, suspended in 0.2% NaCl, heated at 121°C for 30 min, centrifuged at 3,000 rpm for 20 min, and resuspended in 200 μL of 0.2% NaCl. The resuspension was mixed with I/II, V/VI, I, IV, VI, VII, VIII, and IX antisera on a glass slide. Agglutination in the reaction with each serum was regarded as positive for the respective O-antigen. For H-antigen identification, *L*. *monocytogenes* was passed through a Craigie tube 3–4 times in semi-liquid BHI medium (0.2% agar), incubated overnight at 30°C in BHI broth, and suspended in physiological saline containing 1% formalin. Two drops of H-antiserum (A, AB, C, and D) were added to 0.5 mL of each cell suspension and incubated in a constant temperature water bath (50–52°C) for 1 h. Agglutinated samples were determined as positive.

### Molecular serogroup identification

Serogroups of *L. monocytogenes* were determined using the multiplex PCR assay devised by Doumith et al. that targets *lmo0737*, *lmo1118*, *ORF2819*, *ORF2110*, and *prs* (Doumith et al., 2004). A total of 50 μL PCR mixture contained 2 μL template DNA, 25 μL Taq PCR Mastermix (Cosmogenetech, Seoul, South Korea), forward and reverse primers of 1 μL of *lmo0737*, *ORF2819*, and *ORF2110* (50 pmol/μL), 1.5 μL of *lmo1118* (50 pmol/μL) and 1 μL of *prs* (10 pmol/μL) and 12 μL distilled water. Amplification was performed using the T100^™^ Thermal Cycler (Bio-Rad, Hercules, CA, United States). The PCR was run with an initial denaturation step at 94°C for 3 min, followed by 35 cycles of denaturation at 94°C for 40 s, annealing at 53°C for 75 s, and extension at 72°C for 75 s, and the final extension at 72°C for 7 min. PCR amplification products were loaded on a 1.5% agarose gel and visualized under UV illumination (Spectroline model; TVC-312R, Spectronics Corporation, Westbury, NY, United States).

### MLST analysis

MLST was conducted as recommended by the Institut Pasteur^1^ with seven housekeeping genes (*abcZ*, *bglA*, *cat*, *dapE*, *dat*, *ldh*, and *lhkA*). Briefly, PCR started with an initial denaturation step at 94°C for 4 min, followed by 35 cycles consisting of denaturation at 94°C for 30 s, annealing at 52°C (45°C for *bglA*) for 1 min, and extension at 72°C for 2 min, with a final extension at 72°C for 10 min. PCR amplicons were purified using a LaboPass^™^ Gel and PCR Clean-up Kit (Cosmogenetech) and sequenced by Macrogen (Seoul, South Korea). STs and CCs were assigned from the *Listeria* MLST database managed by the Institut Pasteur.^2^ A minimum spanning tree was constructed by using the software GrapeTree (Zhou et al., 2018).

### Antibiotic resistance assays

The food and clinical *L. monocytogenes* isolates were tested for antibiotic resistance using the disk diffusion method as recommended by the Clinical Laboratory Standards Institute (CLSI, 2020). After spreading cells onto the surface of the Muller-Hinton agar (BD, Franklin Lakes, NJ, United States) using a sterile cotton swab, antibiotic paper disks were placed onto the agar plates and incubated at 35°C for 24 h. Paper disks used in this study (Oxoid, Basingstoke, Hampshire, United Kingdom) were impregnated with erythromycin (15 μg), lincomycin (2 μg), amikacin (30 μg), ampicillin (10 μg), trimethoprim (5 μg), penicillin G (10 unit), streptomycin (10 μg), gentamicin (10 μg), tetracycline (30 μg), bacitracin (10 unit) or oxytetracycline (30 μg). The diameter (mm) of the inhibition zone was measured using a digital caliper (CD-20APX; Mitutoyo Corporation, Kanagawa, Japan). The data were interpreted according to the zone diameter breakpoint for *Staphylococcus* spp. since there is no resistance criterion in the CLSI guidelines for *L. monocytogenes* (Carvalho et al., 2019). *S. aureus* ATCC 25923 was used as a quality control strain.

### WGS and the genome comparison

The whole genome of outbreak strain FSCNU0110 was sequenced with MiSeq (Illumina, San Diego, CA, United States) and Oxford Nanopore sequencing technologies (Oxford Nanopore Technologies, Oxford, United Kingdom). For Illumina sequencing, the extracted genomic DNA was fragmented by sonication using a Covaris M220 (Covaris, Woburn, MA, United States). The sheared DNA was then used to prepare a WGS library with an average insert size of 550 bp using a TruSeq Nano DNA Sample Prep kit (Illumina). The library was sequenced on a MiSeq platform with the 300 bp paired-end sequencing mode. Sequencing data were processed to remove low-quality bases and adapter sequences with the optimized settings (LEADING:10 TRAILING:10 SLIDINGWINDOW:4:20 MINLEN:200) using Trimmomatic v0.39 (Bolger et al., 2014). Subsequently, to remove phiX control (Illumina) from pre-assembled data, trimmed sequences were aligned against the phiX genome with bowtie2 v2.3.5.1 (Langmead and Salzberg, 2012) with the default options and filtered out by SAMtools v1.9 (Li et al., 2009). For Nanopore sequencing, a MinION sequencing library was prepared using the Nanopore Ligation Sequencing Kit (SQK-LSK110; Oxford Nanopore Technologies). The library was sequenced with an R9.4.1 MinION flow cell (Flongle; Oxford Nanopore Technologies) for a 24 h run using MinKNOW (Oxford Nanopore Technologies) with the default settings in the MinKNOW core 5.0.0 and Guppy 6.0.6. Sequencing data were basecalled with Guppy basecaller v3.1.5 (Oxford Nanopore Technologies GitHub: Guppy). NanoFilt v2.8.0 (De Coster et al., 2018) filtered out the reads with an average Phred quality score lower than 7 and length lower than 1,000 (parameter: -q < 7). Unicycler v0.4.8 (Wick et al., 2017) was used to construct the whole genome with the combined filtered MiSeq and MinION data. Then, the genome was annotated using Prokka v1.14.6 (Seemann, 2014). FSCNU0110 genome was deposited to GenBank with accession no. CP101619.

The whole genome sequence of FSCNU0110 was compared with the publicly available genome sequence of 23 *L. monocytogenes* strains isolated from South Korea and 12 CC224 strains isolated overseas. For cgMLST analysis, allele distances were compared with 1,748 genes using the Galaxy platform.^3^ The cgMLST profiles were grouped into cgMLST types (CTs) with a cutoff of 7 mismatches and sublineages (SLs) with a cutoff of 150 allele mismatches (Moura et al., 2016). Virulence and antibiotic resistance genes were identified using Genomic Comparator available in the *Listeria* MLST database maintained by the Institut Pasteur.

## Results

### Serotyping and MLST analysis

**Figure 1:**
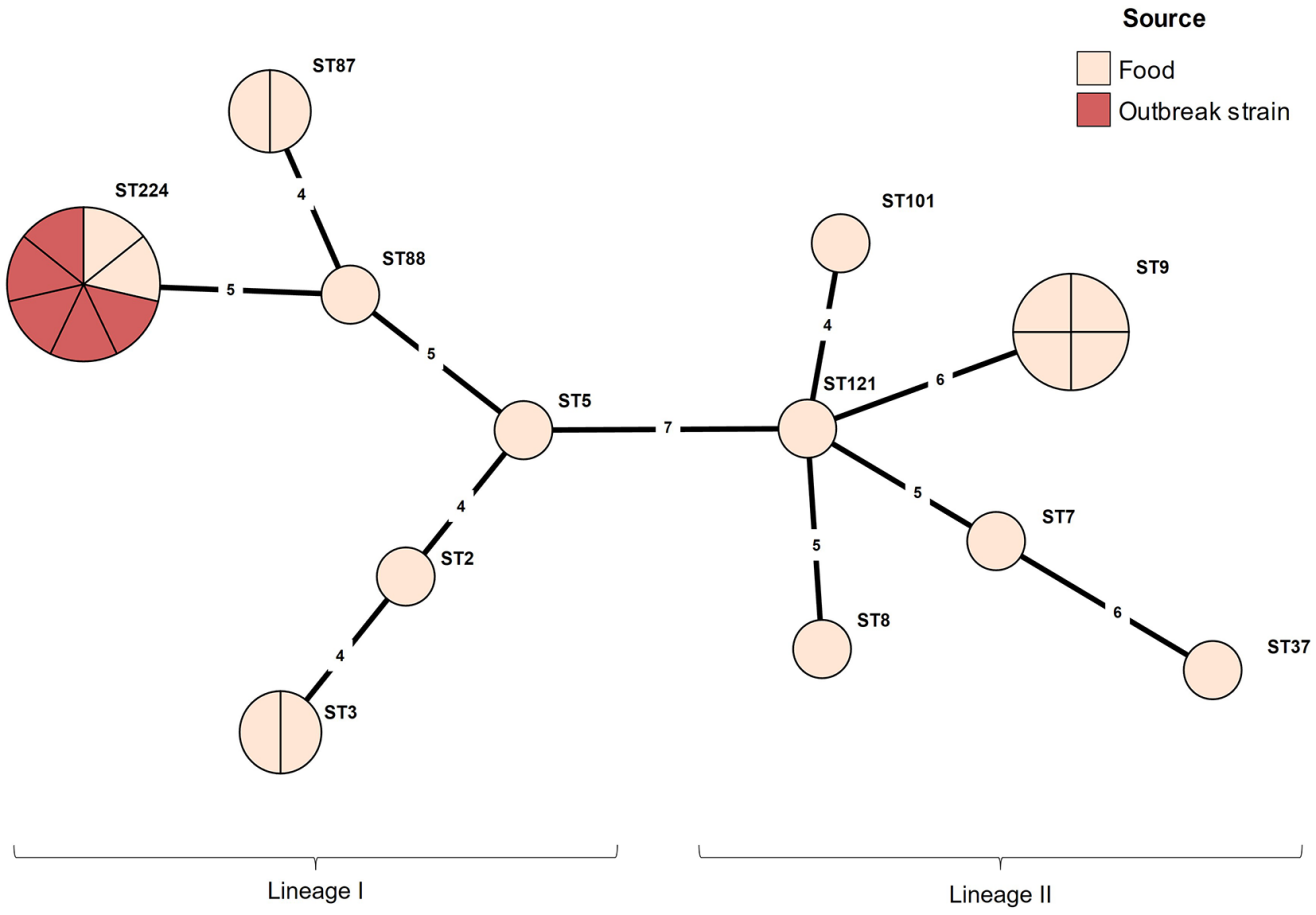
Minimum spanning tree constructed based on MLST allelic profiles of 23 *Listeria monocytogenes* isolates. Each circle clusters a sequence type, and colors distinguish sources. Branch length indicates the difference in the number of alleles.

The serotype and MLST analyses were conducted for the outbreak strain FSCNU0110 and other *L. monocytogenes* isolates from foods (*n* = 19) and humans (*n* = 5) in South Korea (Table 1). Antisera-based serotyping assay revealed that more than half of those isolates belonged to serotype 1/2b (*n* = 14) while three other serotypes [1/2a (*n* = 6), 1/2c (*n* = 4), and 4b (*n* = 1)] were also noted (Table 1). PCR-based serotyping generated consistent results for the serological assay (Table 1). When MLST was conducted, the aforementioned isolates were partitioned into 13 STs, among which ST224 (*n* = 7; lineage I), ST9 (*n* = 4; lineage II), ST3 (*n* = 2; lineage I), ST87 (*n* = 2; lineage I), and ST101 (*n* = 2; lineage II) were identified in multiple isolates while other STs (ST2, ST5, and ST88 belonging to lineage I; ST7, ST8, ST37, ST121, and ST2929 belonging to lineage II) were encountered only once (Table 1 and Figure 1). Clinical isolates were of CC224 (*n* = 4, serotype 1/2b), CC2929 (*n* = 1, serotype 1/2b), and CC101 (*n* = 1, serotype 1/2a) (Table 1 and Figure 1). The outbreak strain FSCNU0110 belonged to ST224, along with two strains isolated from seasoned pork in 2005 (FSCNU0003 and FSCNU0045) (Table 1).

### Antibiotic resistance

Resistance to various antibiotics was tested for *L. monocytogenes* isolated from food and human (Table 2). All these strains exhibited resistance to lincomycin and penicillin G but susceptible to erythromycin, amikacin, trimethoprim, gentamicin, and bacitracin (Table 2). Meanwhile, only a subset of the isolates was fully resistant to ampicillin (60.9%), tetracycline (43.5%), or oxytetracycline (43.5%), or intermediately resistant to streptomycin (56.5%) (Table 2). Interestingly, resistance to tetracycline accompanied oxytetracycline resistance and vice versa (Table 2). Outbreak strain FSCNU0110 showed resistance to ampicillin, tetracycline, and oxytetracycline, which was also observed in other ST224 isolates analyzed in this study (Table 2).

**Table 2:** Antibiotic susceptibility of *L. monocytogenes* isolates.

Isolated	Antibiotics										
	E^a^	MY	AK	AMP	W	P	S	CN	TE	B	OT
FSCNU0002	S^b^	R	S	S	S	R	I	S	R	S	R
FSCNU0003	S	R	S	R	S	R	S	S	R	S	R
FSCNU0007	S	R	S	S	S	R	I	S	R	S	R
FSCNU0045	S	R	S	R	S	R	S	S	R	S	R
FSCNU0052	S	R	S	S	S	R	I	S	S	S	S
FSCNU0057	S	R	S	S	S	R	I	S	S	S	S
FSCNU0060	S	R	S	R	S	R	I	S	S	S	S
FSCNU0062	S	R	S	S	S	R	S	S	S	S	S
FSCNU0066	S	R	S	S	S	R	S	S	S	S	S
FSCNU0089	S	R	S	S	S	R	I	S	R	S	R
FSCNU0093	S	R	S	S	S	R	I	S	S	S	S
FSCNU0095	S	R	S	R	S	R	I	S	S	S	S
FSCNU0098	S	R	S	R	S	R	I	S	S	S	S
FSCNU0099	S	R	S	R	S	R	I	S	S	S	S
iFSCNU0100	S	R	S	S	S	R	S	S	S	S	S
FSCNU0110	S	R	S	R	S	R	S	S	R	S	R
FSCNU0111	S	R	S	R	S	R	S	S	R	S	R
FSCNU0113	S	R	S	R	S	R	S	S	R	S	R
FSCNU0114	S	R	S	R	S	R	S	S	R	S	R
FSCNU0115	S	R	S	R	S	R	S	S	R	S	R
FSCNU0120	S	R	S	R	S	R	I	S	S	S	S
FSCNU0121	S	R	S	R	S	R	I	S	S	S	S
FSCNU0122	S	R	S	R	S	R	I	S	S	S	S
NCCP14714	S	R	S	R	S	R	R	S	R	S	R
NCCP15743	S	R	S	S	S	R	R	S	S	S	S

^a^E, Erythromycin (15 μg); MY, lincomycin (2 μg); AK, amikacin (30 μg); AMP, ampicillin (10 μg); W, trimethoprim (5 μg); P, penicillin G (10 unit); S, streptomycin (10 μg); CN, gentamicin (10 μg); TE, tetracycline (30 μg); B, bacitracin (10 unit); and OT, oxytetracycline (30 μg).

^b^S, susceptible; I, intermediate resistance; Rs resistant.

### WGS-based characterization of the outbreak strain FSCNU0110

**Figure 2:**
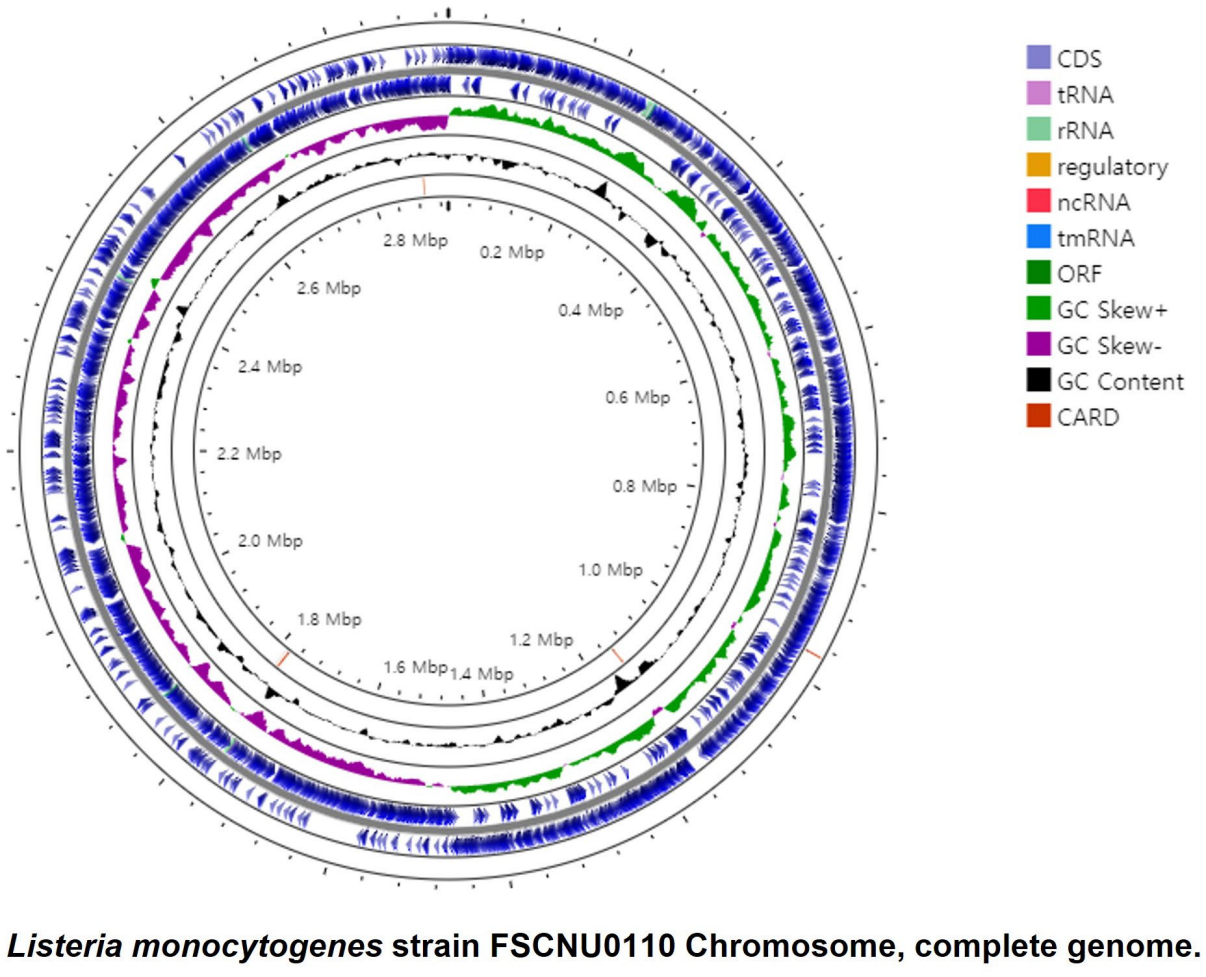
Circular genome map of the *L. monocytogenes* FSCNU0110 chromosome. The genome is 2,933,635, with an average GC content of 37.99%. The circular genome was generated with Proksee (https://proksee.ca/).

FSCNU0110 possessed a single chromosome of 2,982,685 bp with a G + C content of 37.99% that contains 2,913 genes, including 2,824 coding sequences (CDS), 67 tRNA genes, 18 rRNA genes, and 4 ncRNA genes (Figure 2). *In silico* analysis of this genome sequence supported the data obtained with the serological and PCR serotyping assays, and MLST revealed that this strain belonged to SL6178. Five antibiotic resistance genes were identified in this genome via Genome Comparator: *fosX* (fosfomycin), *norB* (quinolone), *sul* (sulfonamides), *lin* (lincomycin), and *tetM* (tetracycline). Also, 64 virulence genes were found, including *Listeria* pathogenicity island 1 (LIPI-1) (*prfA*, *plcA*, *hly*, *mpl*, *actA*, *plcB*), eight *lls* genes in LIPI-3, and 10 internalin genes. No premature stop codons (PMSCs) were detected in *inlA* (Jacquet et al., 2004).

### Comparison of FSCNU0110 with CC224 strains

**Figure 3:**
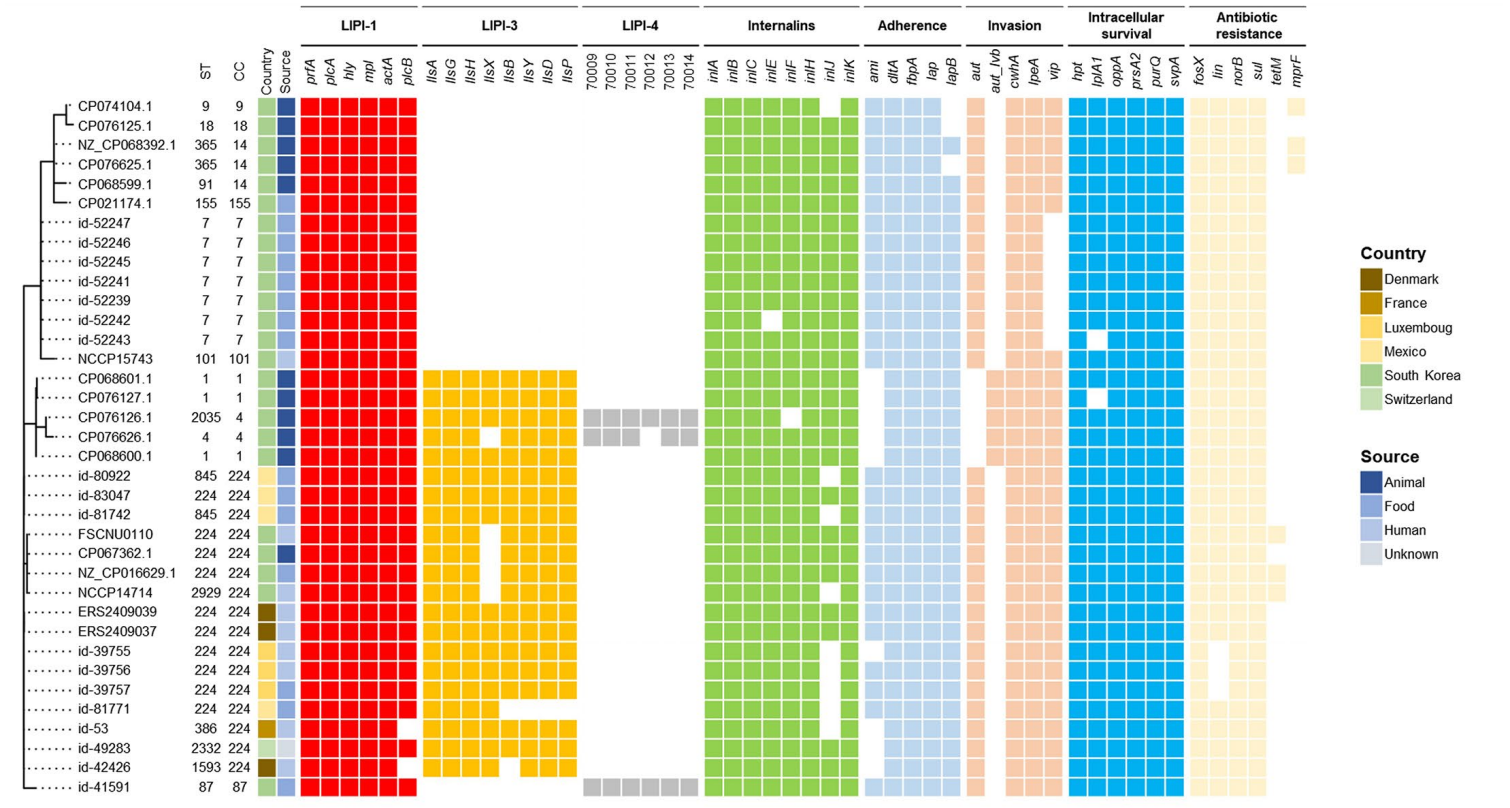
Phylogenetic MLST and comparison of virulence genes and antibiotic resistance genes. The foodborne outbreak strain obtained in this study was compared with the publicly available *L. monocytogenes* genome sequence isolated from Korea and the CC224 genome sequence known internationally, indicating the presence (color squares) or absence of a gene (white square).

**Figure 4:**
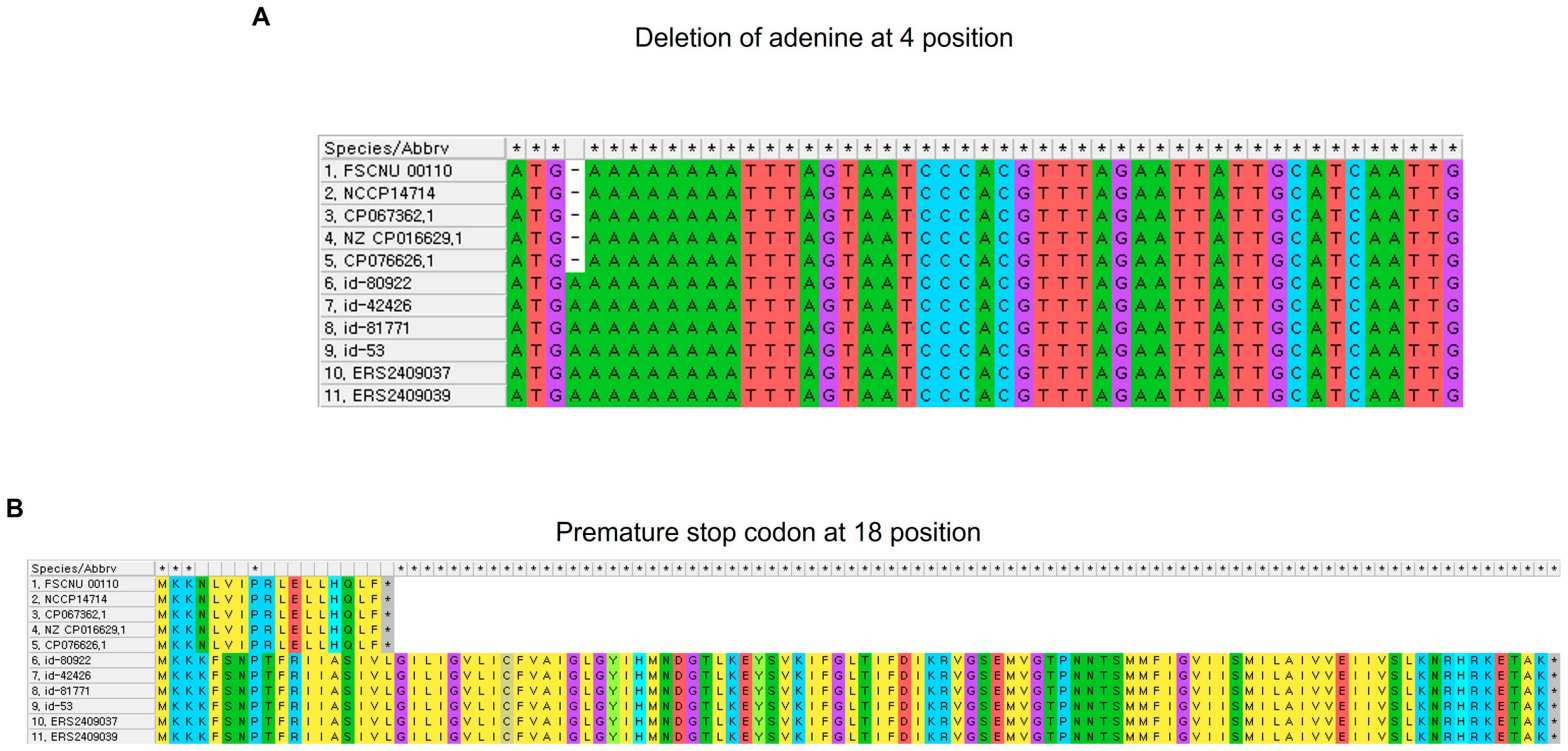
Adenine deletion identified in the *llsX* gene in *L. monocytogenes*. **(A)** Nucleotide alignment, and **(B)** amino acid alignment.

The genome of the outbreak strain FSCNU0110 was compared with *L. monocytogenes* strains isolated from South Korea and CC224 strains isolated overseas, whose genomes were available in the NCBI and Institut Pasteur MLST database, regarding the virulence and antibiotic resistance genes (Figure 3). While *inlA* was highly conserved without any PMSC, LIPI-3 was only harbored in a subset of lineage I, including all CC224 strains regardless of geographical origin, and LIPI-4 was found in only three strains belonging to CC4 and CC87. When the LIPI-3 sequences were compared between overseas CC224 strains and those from South Korea, one adenine deletion at position 4 of *llsX* (allele 1) was found in outbreak strain FSCNU0110 and other domestic CC224 strains, which resulted in a PMSC at position 18, producing a 17-amino acid fragment (Figure 4). In addition, strain CP076626.1 in ST4 and CC4 isolated from South Korea had the same adenine deletion in *llsX* (allele2) with PMSC (Figures 3, 4). Regarding five antibiotic resistance genes that were identified in FSCNU0110, *fosX*, *norB*, *sul* and *lin* were harbored by all the strains except that three strains from Luxembourg (id-39755, id-39756, and id-39757) were missing *lin* (Figure 3). On the other hand, *tetM* was found only among three domestic CC224 strains (FSCNU0110, FORC_049, and NCCP14714).

Allele differences based on cgMLST are shown in Table 3. The strains with less than 150 allele differences were categorized as the same CC strains, while the allele differences of more than 1,000 were identified for different CC strains. In the CC224, there were 18–48 allele differences among the domestic CC224 strains, including FSCNU0110, and 2–100 allele differences among the overseas CC224 strains. However, 325–407 allele differences were found between domestic and overseas strains of CC224. Our cgMLST analysis revealed that CC224 was regionally segregated since the overseas strains belonged to SL224 whereas the domestic strains, including FSCNU0110, were classified into SL6178. Additionally, there were 18–48 allele differences between the domestic strains of CC224 and FSCNU0110.

**Table 3:** Allele differences based on cgMLST.

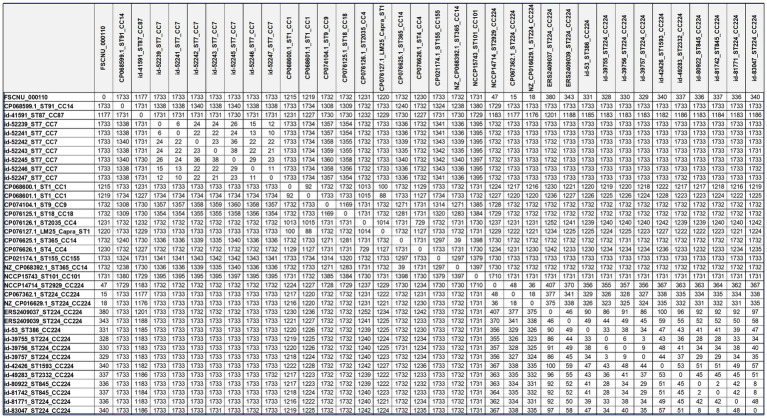

## Discussion

In this study, we characterized *L. monocytogenes* strain FSCNU0110 involved in the first listeriosis foodborne outbreak in South Korea employing a WGS strategy. The high-accuracy sequences were obtained with Illumina technology, and the inter-contig gaps were filled with long-read sequences generated with Oxford Nanopore technology, thus enabling us to obtain the complete genome of this strain. This outbreak strain belonged to ST224 (serotype 1/2b), which was also isolated in listeriosis patients in 2009 in a survey conducted in a hospital in South Korea and has been obtained from several foods collected from different countries (De Cesare et al., 2017; Choi et al., 2018; Lake et al., 2021). Recently, ST224 caused an outbreak implicating delicatessen meat in Denmark, resulting in 41 cases and 17 deaths (Kvistholm et al., 2016).

In addition to the outbreak strain FSCNU0110, we also included in the analysis multiple strains (*n* = 19) of *L. monocytogenes* strains isolated from foods sold in South Korea. These included all the major serotypes of *L. monocytogenes* involved in clinical cases and frequently isolated from foods and environment (1/2a, 1/2b, 1/2c, and 4b) (Swaminathan and Gerner-Smidt, 2007; Kramarenko et al., 2013; Zhang et al., 2019). Notably, these food isolates included eight STs that were encountered in the aforementioned survey conducted with listeriosis patients in a local hospital in South Korea between 2009 and 2016 (Choi et al., 2018), suggesting that these STs in the foods might have been causing listeriosis in South Korea. A majority of these STs were previously reported from a meat processing plant in Spain (Martín et al., 2014), RTE meat, vegetables, and cooked foods sold in China (Wang et al., 2018).

LIPI-3 is a pathogenicity island harbored by a subset of lineage I and produces listeriolysin S (LLS), a bacteriocin that modulates the gut microbiota composition to favor infection (Cotter et al., 2008; Clayton et al., 2014; Quereda et al., 2017). LLS is a cytolytic peptide produced and modified post-translationally by the *lls* operon composed of eight genes (Clayton et al., 2014; Quereda et al., 2017). *llsA* is a gene encoding a structural peptide; *llsB*, *llsY*, and *llsD* genes are for post-translational modification enzymes; *llsG* and *llsH* genes encode a putative ABC transporter; and *llsP* gene is annotated as a putative protease (Cotter et al., 2008). However, the function of the *llsX* gene has yet been identified (Quereda et al., 2017). The *llsX* gene was highly conserved and utilized to detect LIPI-3 (Chen et al., 2019). However, in CC224 strains from South Korea, the *llsX* gene had the adenine deletion in location 4, resulting in a PMSC. This result raised the possibility of using this SNP as a genetic marker to distinguish domestic CC224 strains from their overseas counterparts. As reported for *inlA* PMSC that attenuated the virulence of *L. monocytogenes* (Jacquet et al., 2004; Holch et al., 2013; Maury et al., 2016), although the physiological role of the protein encoded in the *llsX* gene, this mutation in the *llsX* gene could impact the pathogenicity of *L. monocytogenes*, and further research is warranted.

In strains FSCNU0110 and NCCP14714, we found genes that confer resistance to lincomycin (*lin*) and tetracycline (*tetM*), and these two strains were indeed strongly resistant to these antibiotics. Lincomycin and tetracycline are used for clinical practice to inhibit protein synthesis in bacteria. Previous studies showed resistance to lincomycin in *L. monocytogenes* isolated from foods in Italy (100%) and food processing environments in China (90%) (Mauro et al., 2007; Wu et al., 2021). Lincomycin inhibits protein synthesis by binding to the 50S ribosomal subunit (Papich, 2016). *L. monocytogenes* shifts the riboregulator Rli53 construct from a closed state to an open state leading to the expression of *lmo0919* (*lin*), which confers resistance to lincomycin (Radoshevich and Cossart, 2018). The *tetM* gene is the most prevalent tetracycline resistance gene in *Listeria* species (Charpentier et al., 1995; Bertrand et al., 2005). Tetracycline inhibits protein synthesis by binding the 30S ribosomal subunit, while the ribosome protective protein (TetM) binds to the ribosome and confers resistance to tetracycline by chasing the drug at the binding site (Arenz et al., 2015). Previous studies observed resistance to tetracycline in approximately one-fifth of *L. monocytogenes* isolates from ready-to-eat seafood and food processing environments in South Korea (Lee et al., 2017). Additionally, ampicillin resistance was noted for the outbreak strain FSCNU0110. All these findings suggest that antibiotic resistance phenotypes of *L. monocytogenes* strains isolated from South Korea should be considered when administering an antibiotic for listeriosis patients.

The clustering efficiency of identical sublineages was optimal with 140–150 allele differences, and a threshold of 150 allele differences (8.58% dissimilarity) was chosen to define sublineages. Isolates belonging to different phylogenetic lineages differed in 1,500 allele differences among 1,748 loci, and isolates belonging to different sublineages among isolates belonging to the same phylogenetic lineage showed 1,000–1,400 allele differences. In addition, the cgMLST-based sublineage designations were mapped with the 7-MLST-based CCs. In the case of CC224, 54 *L. monocytogenes* strains isolated in the United States, France, Denmark, and the United Kingdom were identified as SL224 (Moura et al., 2016). The SL of CC224 stains confirmed in North America, and Europe was also consistent in our study as SL224. Only the domestic strain of CC224 was classified into SL6178, indicating the allele differences by their regional characteristics, that FSCNU0110 and SL6178 of CC224 suggest the possibility of being index of domestic strains in South Korea. For a more accurate and sophisticated investigation of the source of contamination, the whole genome-based examination will need to be implemented for domestic and imported food products, as exemplified by the multi-state listeriosis outbreak in 2016–2019 implicating enoki mushrooms that was noted four years after the first case owing to the comparison of WGS data retrieved from the PulseNet (Pettengill et al., 2020).

In this study, the genome sequence of the outbreak strain was analyzed and compared with publicly available CC224 strains of overseas and domestic origins, which included a limited number of the local strains (*n* = 3) and no strains from other Asian countries. The comparative analysis should include CC224 strains from various isolation sources and geographical regions to obtain reliable epidemiological data. Also, listeriosis cases have been continuously reported in South Korea, but only two human isolates have been genome-sequenced. As listeriosis cases continue to occur and *L. monocytogenes* is isolated from food distributed in Korea, epidemiological data such as WGS in Korea should be further studied.

## Conclusion

Our analysis of the WGS data of the *L. monocytogenes* strain involved in the first listeriosis foodborne outbreak reported in South Korea revealed several genetic features shared by this strain and other *L. monocytogenes* strains collected from South Korea, which could differentiate these local isolates from those collected outside South Korea. Further studies on the characteristics of South Korea-originating *L. monocytogenes* strains will be required to implement detection and control measures better suited to the local *L. monocytogenes* population.

## Data availability statement

The datasets presented in this study can be found in online repositories. The names of the repository/repositories and accession number(s) can be found at: https://www.ncbi.nlm.nih.gov/genbank/, CP101619.1.

## Author contributions

OK and SP conceived, designed, reviewed, and managed the study. SHL conducted the experiments and contributed to data analysis, manuscript drafting, and drawing. SL analyzed and interpreted the data and contributed to the writing of the manuscript text. All authors contributed to the manuscript and read and approved the final version.

## Funding

This research was supported by the National Research Foundation of Korea (NRF-2022R1A4A1033015 and NRF-2019R1C1C1002427).

## Conflict of interest

The authors declare that the research was conducted in the absence of any commercial or financial relationships that could be construed as a potential conflict of interest.

## Publisher’s note

All claims expressed in this article are solely those of the authors and do not necessarily represent those of their affiliated organizations, or those of the publisher, the editors and the reviewers. Any product that may be evaluated in this article, or claim that may be made by its manufacturer, is not guaranteed or endorsed by the publisher.

## Footnotes

^1^ https://bigsdb.pasteur.fr/listeria/primers-used/

^2^ https://bigsdb.pasteur.fr/listeria/

^3^ https://galaxy.sciensano.be
